# Impact of the timing of tumor assessments on median progression-free survival in clinical trials in advanced cancer patients

**DOI:** 10.1016/j.esmoop.2021.100366

**Published:** 2021-12-31

**Authors:** T. Samaille, C. Moreau Bachelard, E. Coquan, P. du Rusquec, X. Paoletti, C. Le Tourneau

**Affiliations:** 1 Department of Drug Development and Innovation (D3i), Institut Curie, Paris, France; 2 Sorbonne Université, Faculté de médecine, Paris, France; 3 Department of Medical Oncology, Institut de Cancerologie de l’Ouest, Boulevard Jacques Monod, Nantes Saint-Herblain, France; 4 Department of Medical Oncology, Centre François Baclesse, Caen, France; 5 INSERM U900 Research Unit, Saint-Cloud, France; 6 Paris-Saclay University, Gif-sur-Yvette, France

**Keywords:** clinical trials, evaluation-time bias, progression-free survival, timing assessment, meta-analysis

## Abstract

**Background:**

Survival-based surrogate endpoints such as progression-free survival (PFS) are commonly used in oncology clinical trials. The evaluation-time bias in the assessment of median disease progression in randomized trials has been suggested by several simulation studies, but never demonstrated in the clinic. We aimed to demonstrate the existence of potential evaluation-time bias by assessing the impact of the timing of tumor assessments on median PFS from control arms without any active treatment of randomized controlled trials involving advanced cancer patients.

**Materials and methods:**

A systematic literature search of English language publications from 1 January 2000 to 7 January 2021 was performed using MEDLINE (PubMed). Eligible trials for our meta-analysis included all randomized clinical trials evaluating anticancer drugs in adult patients with advanced cancers with a control arm without any anticancer drug consisting of best supportive care with or without a placebo. We performed a meta-regression analysis to analyze the correlation between the timing of the first tumor assessment and median PFS in patients randomized in the control arms without any active treatment.

**Results:**

Of 3551 studies screened, 97 eligible trials were retrieved involving 36  747 patients, including 14  229 patients randomized into the control arms. A later first tumor assessment correlated with a prolonged median PFS (R^2^ = 0.44, *P* < 10^−5^).

**Conclusions:**

Our results confirm the existence of potential evaluation-time bias in clinical research that had been suggested by simulation studies. The timing of tumor assessments should be kept the same in precision medicine trials using the PFS ratio as an efficacy endpoint.

## Highlights

• Different timings for the evaluation of efficacy can induce an evaluation-time bias as suggested by simulation studies.
• This bias can be an important issue in precision medicine trials that use each patient as their own controls.
• In our meta-analysis, we found a correlation between the timing of the first tumor assessment and median PFS.
• Our study confirms and quantifies the evaluation-time bias suggested by simulation studies.
• Our study supports having timings of tumor assessments be kept the same in precision medicine trials.

## Introduction

The primary objective of any intervention in oncology is to improve overall survival and/or quality of life. Surrogate endpoints of survival such as the overall response rate and progression-free survival (PFS) are commonly used with the aim to get an early read-out for go/no go decisions and/or speeding up market access. The estimation of these surrogate endpoints mostly relies on standardized criteria, including the World Health Organization (WHO) criteria and RECIST.^1^^,^^2^

A more comprehensive understanding of cancer biology has led to the development of molecularly targeted agents that trigger specific molecular alterations. Trials evaluating such anticancer agents in molecularly driven cohorts of patients are commonly named ‘precision medicine trials’.

Several simulation studies have corroborated an obvious intuition that the timing of tumor assessments might affect the measure of PFS.^3^, ^4^, ^5^ Different timings of tumor assessments across arms of randomized clinical trials can induce this well-described evaluation-time bias. Recommendations have been made to minimize this bias in randomized controlled clinical trials.^6^ This evaluation-time bias can be an important issue when estimating median PFS in single-arm trials and in precision medicine trials that use each patient as his/her own control. Several precision medicine trials evaluated the ratio of the PFS on matched therapy to the PFS on last received treatment in each individual patient to determine the efficacy of matched therapy.^7^, ^8^, ^9^, ^10^, ^11^, ^12^

Since the evaluation-time bias has only been estimated using simulations, we aimed to demonstrate the existence of this bias by performing a systematic review and meta-analysis of the control arms without any active anticancer drug from randomized clinical trials in the recurrent and/or metastatic setting, and to evaluate the impact of the timing of tumor assessments on median PFS in the control arms.

## Material and methods

### Studies selection and data collection

Eligible trials for our meta-analysis included all randomized clinical trials evaluating anticancer drugs in adult patients with advanced cancers with a control arm without any anticancer drug consisting of best supportive care with or without a placebo. Clinical trials performed in the adjuvant, neoadjuvant, or maintenance settings were excluded, as were clinical trials evaluating anticancer drugs in combination with radiotherapy. This systematic review was conducted according to Preferred Reporting Items for Systematic Review and Meta-Analyses (PRISMA) guidelines.^13^

To retrieve these trials, a MEDLINE search was performed from 1 January 2000 to 7 January 2021 using the following search terms: ‘placebo OR best supportive care AND cancer AND controlled randomized trial AND survival’. The National Institutes of Health (NIH) US National Library of Medicine was also searched through ClinicalTrials.gov using the keywords ‘placebo controlled’ OR ‘best supportive care controlled’, ‘completed’, ‘terminated studies’, ‘interventional studies’, ‘advanced cancer’, ‘phase 2, 3’ to identify missing trials. Abstracts of references that appeared potentially eligible for inclusion were examined independently by two reviewers (CMB and EC) and, if deemed relevant, full-text articles including supplementary materials were retrieved and included if appropriate. Disagreements between the two reviewers were resolved by consensus with one of us (CLT). Only papers published in the English language were considered.

Trial characteristics included primary tumor location, line of therapy, type of anticancer drug evaluated in the experimental arm, phase of the clinical trial, number of patients in each arm, and planned timing of tumor assessments. Cancer types were grouped into seven categories based on the classification used by the European Society of Medical Oncology.^14^

### Statistical analysis

In a meta-regression, we assessed the correlation between the median PFS and the planned timing of first tumor assessment weighted on trials’ number of patients and adjusted on the tumor category, and the number of previous lines of treatment. Influence of the timing of the first tumor assessment was estimated through the regression coefficient. Linearity was assessed through visual analysis of residuals. All statistical analyses were performed using R software version 3.3.3.

### Data availability

The study protocol is registered in the International Prospective Register of Systematic Reviews (PROSPERO; CRD42021243968). Raw data are available upon request.

## Results

**Figure 1:**
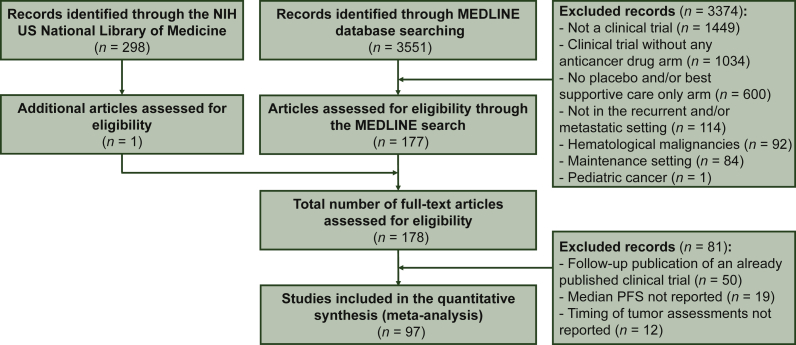
**Study selection process of randomized clinical trials with a control arm without an active drug.** NIH, National Institutes of Health.

We retrieved 97 trials published between 1 January 2000 and 7 January 2021 that matched our selection criteria (Figure 1 and Table 1). A total of 36  747 patients were included in these trials, including 14  229 patients (38.7%) randomized into the control arms. The median timing for the first tumor assessment was week 8 (range, week 3-16). RECIST was used in 87 out of the 97 trials (89.7%) to assess PFS.

**Table 1:** Characteristics of the trials

Characteristics	Values, *n* (%)
Trials, *n*	97
Patients, *n*	36 747
Experimental arm	22 518 (61.3)
No active treatment arm	14 229 (38.7)
Cancer categories	
Gastrointestinal	38 (39.2)
Lung	20 (20.6)
Genitourinary	17 (17.5)
Neuroendocrine	11 (11.3)
Sarcoma	9 (9.3)
Melanoma	1 (1.0)
Head and neck	1 (1.0)
Criteria used for efficacy assessment	
RECIST	87 (89.7)
World Health Organization (WHO) criteria	6 (6.2)
Other	2 (2.1)
Not specified	2 (2.1)
Timing of first efficacy assessment	
Week 3	1 (1.0)
Week 4	13 (13.4)
Week 5	2 (2.1)
Week 6	31 (32.0)
Week 7	1 (1.0)
Week 8	29 (29.9)
Week 9	1 (1.0)
Week 10	1 (1.0)
Week 12	14 (14.4)
Week 13	1 (1.0)
Week 15	1 (1.0)
Week 16	2 (2.1)
Clinical phase of the trial	
Phase 2	29 (29.9)
Phase 3	68 (70.1)
Experimental treatment type	
Chemotherapy	12 (12.4)
Hormone therapy	9 (9.3)
Molecularly targeted agent	66 (68.0)
Immunotherapy	10 (10.3)
Number of prior lines of treatment	
0-1	56 (57.7)
≥2	41 (42.3)

**Figure 2:**
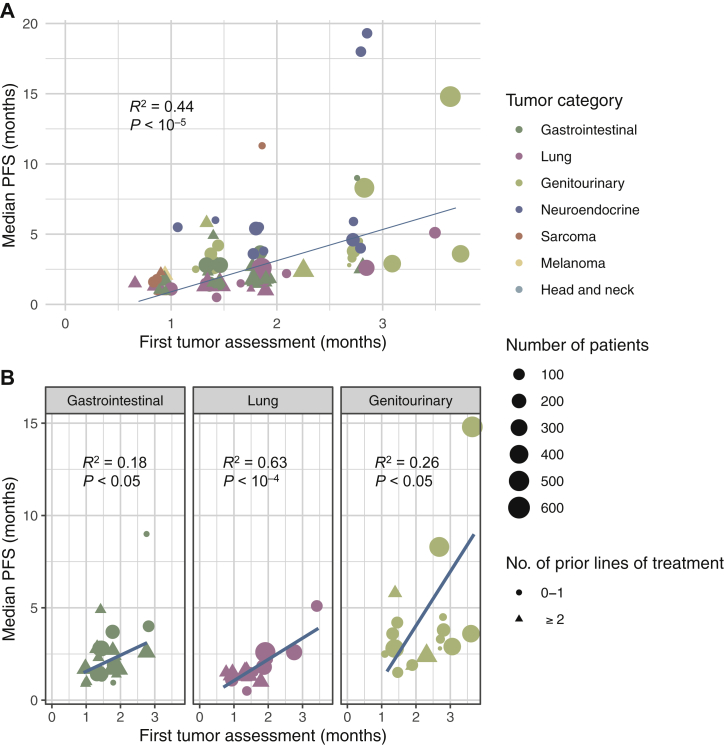
**Regression analysis for median PFS in (A) all studies, and (B) three most represented tumor categories. The blue lines are the regression lines.** PFS, progression-free survival.

Overall, the timing of the first tumor assessment correlated with the median PFS (R^2^ = 0.44, *P* < 10^−5^; Figure 2A). The regression coefficient was 2.22 (95% confidence interval [CI]: 1.38–3.06, *P* < 10^−5^), meaning that delaying the first tumor assessment by 1 month would increase the median PFS by an average of 2.22 months.

The correlation was stronger for thoracic malignancies (R^2^ = 0.63, *P* < 10^−4^) than for gastrointestinal (R^2^ = 0.18, *P* < 0.05) and genitourinary (R^2^ = 0.26, *P* < 0.05) tumors (Figure 2B). The regression coefficient were 2.82 (95% CI 0.66-4.97, *P* = 0.02) for genitourinary tumors, 1.08 (95% CI 0.63-1.52, *P* < 0.01) for thoracic malignancies, and 0.95 (95% CI 0.30-1.59, *P* < 0.01) for gastrointestinal tumors.

The correlation between the timing of first tumor assessment and PFS was not impacted by trial phase [regression coefficient of 0.10 (95% CI −1.71 to 1.92), *P* = 0.9] nor by treatment line [regression coefficient of −0.42 (95% CI −1.65 to 0.81), *P* = 0.5].

## Discussion

Our results definitely confirm the existence of potential evaluation-time bias in clinical research that had been suggested by simulation studies. In the control arms of randomized controlled trials versus no active treatment, the timing of tumor assessments strongly correlated with the median PFS. Tumor assessments performed in this untreated patient population reflect the natural history of cancer in the recurrent and/or metastatic setting without being impacted by an anticancer therapy, in contrast to simulations studies that were mostly derived from treatment arms from randomized controlled trials.^3^, ^4^, ^5^

The strongest correlation with PFS was found for thoracic malignancies, as compared with gastrointestinal and genitourinary malignancies. This result might likely be explained by the fact that thoracic malignancies represented a more homogeneous group of diseases than the gastrointestinal and the genitourinary groups. Indeed, 16 out of the 20 studies involving thoracic malignancies included patients with non-small-cell lung cancer, whereas gastrointestinal studies included different cancer types known to have different prognoses [hepatocellular carcinoma (*n* = 17), colorectal cancer (*n* = 12), gastric cancer (*n* = 6), cholangiocarcinoma (*n* = 2), and pancreatic cancer (*n* = 1)]. Similarly, genitourinary trials included cancer types with established varied prognoses [prostate cancer (*n* = 10), renal cell cancer (*n* = 5), and bladder cancer (*n* = 2)].

Recommendations had been made regarding the timing of tumor assessments in randomized clinical trials, with a key message being to have similar timings in all treatment arms, in order to get unbiased estimate of the hazard ratio whatever the timing is.^6^ While it is elusive to prone harmonization of these timings to be able to compare median PFS results across trials including similar patient populations, different timings for tumor assessments is one additional challenge for such intertrial comparisons.

Because of the molecular segmentation of cancer and the discovery of rare alterations that might be relevant across cancer types, precision medicine trials that mix cancer types, molecular alterations, and therapies have become more common. While the trials are infrequently randomized,^8^ most of them have used the PFS ratio to individually evaluate the efficacy of matched therapy as compared with standard therapy.^7^^,^^9^, ^10^, ^11^, ^12^ In these trials, median PFS on matched therapy was short, ranging from 2.0 to 3.7 months. Our results underline the absolute necessity of using the same timings of tumor assessments on both treatments for a same patient, to avoid a substantial evaluation-time bias. As an example, the ongoing SHIVA02 precision medicine trial has been designed with both PFS being assessed with tumors assessments every 2 months using RECIST (NCT01771458).

Our study has several limitations, the first one being that we had no individual patient data. We then correlated here the planned timing of the first tumor assessment with the median PFS, but did not know when the first tumor assessment actually occurred. Finally, PFS was always analyzed assuming that progression occurred at the date of assessment, while it certainly happened between the evaluation dates (interval censored observation). Median PFS is likely to be over-estimated.^5^

### Conclusions

Our results confirm the existence of potential evaluation-time bias for the evaluation of the PFS that had been suggested by simulation studies. The timing of tumor assessments should be kept the same in precision medicine trials using the PFS ratio as an efficacy endpoint.

## References

[1] Therasse P., Arbuck S.G., Eisenhauer E.A. (2000). New guidelines to evaluate the response to treatment in solid tumors. European Organization for Research and Treatment of Cancer, National Cancer Institute of the United States, National Cancer Institute of Canada. J Natl Cancer Inst.

[2] Eisenhauer E.A., Therasse P., Bogaerts J. (2009). New response evaluation criteria in solid tumours: revised RECIST guideline (version 1.1). Eur J Cancer.

[3] Kay R., Wu J., Wittes J. (2011). On assessing the presence of evaluation-time bias in progression-free survival in randomized trials. Pharm Stat.

[4] Filleron T., Kouokam W., Gilhodes J. (2016). Statistical controversies in clinical research: should schedules of tumor size assessments be changed?. Ann Oncol.

[5] Panageas K.S., Ben-Porat L., Dickler M.N., Chapman P.B., Schrag D. (2007). When you look matters: the effect of assessment schedule on progression-free survival. J Natl Cancer Inst.

[6] Dancey J.E., Dodd L.E., Ford R. (2009). Recommendations for the assessment of progression in randomised cancer treatment trials. Eur J Cancer.

[7] von Hoff D.D., Stephenson J.J., Rosen P. (2010). Pilot study using molecular profiling of patients’ tumors to find potential targets and select treatments for their refractory cancers. J Clin Oncol.

[8] Le Tourneau C., Delord J.-P., Gonçalves A. (2015). Molecularly targeted therapy based on tumour molecular profiling versus conventional therapy for advanced cancer (SHIVA): a multicentre, open-label, proof-of-concept, randomised, controlled phase 2 trial. Lancet Oncol.

[9] Massard C., Michiels S., Ferté C. (2017). High-throughput genomics and clinical outcome in hard-to-treat advanced cancers: results of the MOSCATO 01 trial. Cancer Discov.

[10] Sicklick J.K., Kato S., Okamura R. (2019). Molecular profiling of cancer patients enables personalized combination therapy: the I-PREDICT study. Nat Med.

[11] Belin L., Kamal M., Mauborgne C. (2017). Randomized phase II trial comparing molecularly targeted therapy based on tumor molecular profiling versus conventional therapy in patients with refractory cancer: cross-over analysis from the SHIVA trial. Ann Oncol.

[12] Rodon J., Soria J.-C., Berger R. (2019). Genomic and transcriptomic profiling expands precision cancer medicine: the WINTHER trial. Nat Med.

[13] Moher D., Liberati A., Tetzlaff J., Altman D.G., PRISMA Group (2009). Preferred Reporting Items for Systematic Reviews and Meta-Analyses: the PRISMA statement. Ann Intern Med.

[14] https://www.esmo.org/guidelines.

